# High-temperature alert: PHYTOCHROME INTERACTING FACTOR 4 regulates microtubule organization to mediate high temperature-induced hypocotyl elongation

**DOI:** 10.1093/plcell/koad068

**Published:** 2023-03-09

**Authors:** Arpita Yadav

**Affiliations:** Assistant Features Editor, The Plant Cell, American Society of Plant Biologists, USA; Biology Department, University of Massachusetts Amherst, MA 01003, USA

Some plants can acclimate to changing environmental temperatures by changing their morphology. For example, during periods of high ambient temperature, plants undergo thermomorphogenesis, which includes elongation of hypocotyls, stems, and petioles, and leaf curling known as hyponasty. These morphological changes improve leaf cooling and help protect the photosynthetic machinery from heat stress (Casal and Balasubramanian 2019; Delker et al. 2023).

The bHLH transcription factor PHYTOCHROME-INTERACTING FACTOR 4 (PIF4) is a key component controlling hypocotyl length during thermomorphogenesis. Briefly, photoreceptor phytochrome B (phyB) has been shown to be a thermosensor in plants. Active phyB inhibits PIF4 function and promotes its degradation. Elevated ambient temperature converts active phyB to its inactive configuration, which results in PIF4 stabilization. PIF4 activates auxin biosynthetic genes like *YUCCA8* (Sun et al. 2012). Auxin then initiates a signaling cascade to promote cell elongation genes. Although PIF4 had previously been implicated in high temperature-induced hypocotyl elongation through auxin, it is still unclear how PIF4 regulates downstream cellular effectors that may be directly involved in hypocotyl elongation.

Microtubules are known for orienting the cellulose microfibrils to build the mechanical properties of the cell wall that control cell growth. The organization of cortical microtubules is linked to the growth status of hypocotyl cells: transversely oriented cortical microtubules promote rapid growth of hypocotyl cells, whereas longitudinally oriented microtubules slow down hypocotyl cell elongation (Bashline et al. 2014). Previous work suggested that microtubule remodeling in response to changing temperatures may be mediated by MICROTUBULE-ASSOCIATED PROTEINS (MAPs). Among these microtubule-localized proteins, SPIRAL1 (SPR1) had been shown to be required for maintenance of growth anisotropy in elongating cells (Nakajima et al., 2004). However, the underlying mechanism of how microtubules respond to high temperatures to regulate hypocotyl growth remained unclear.

In this issue of *The Plant Cell*, Zhou et al. (2023) screened known mutants of *MAPs* for defects in response to high ambient temperature (28°). Among *map65-1*, *wdl4*, *wdl5*, *spr1*, and *mdp60* mutants screened, they found that the *spr1* mutants showed shorter hypocotyl and petiole lengths at 28°, suggesting that SPR1 promotes high temperature-induced morphological changes. Increasing the growth duration at 28° increased predominantly the formation of transverse cortical microtubules in wild type but not in *spr1* mutants, indicating that SPR1 is a critical MAP in microtubule reorganization at high ambient temperature. Additional experiments revealed that high temperature stimulated the expression of *SPR1* in a PIF4-dependent manner, which correlated with increased SPR1 abundance.

Yeast-2-hybrid screening identified (Plant U-Box-type E3 Ubiquitin Ligase) PUB31 as a potential SPR1 interacting partner, and follow-up experiments showed that PUB31 can ubiquitinate and promote the degradation of SPR1. The authors further found that SPR1 degradation via PUB31 was suppressed under high temperatures because PIF4 directly binds to the *PUB31* promoter and represses its expression at high ambient temperature. In addition, reduced PUB31 abundance results in increased SPR1 stability and hypocotyl elongation at 28 °C. Concomitantly, microtubule reorganization from longitudinal to transverse orientation was accelerated in *pub31* mutant hypocotyl cells at 28°. Hence, PUB31 negatively regulates high ambient temperature-induced hypocotyl elongation and microtubule reorganization.

High temperature often accompanies drought and salinity in soils. Based on a previous report of salt stress-induced degradation of SPR1 (Wang et al. 2011), the authors found that SPR1 degradation and microtubule disassembly were suppressed in *pub31* mutants in response to salt stress, implicating the role of the SPR1-PUB31 module in changing microtubule orientation and assembly in response to other environmental signals. In their thorough study, the authors have thus identified a new mechanism whereby PIF4 fine-tunes the SPR1-PUB31 module to reorganize microtubules in response to high temperature and other developmental signals.

**Figure.**
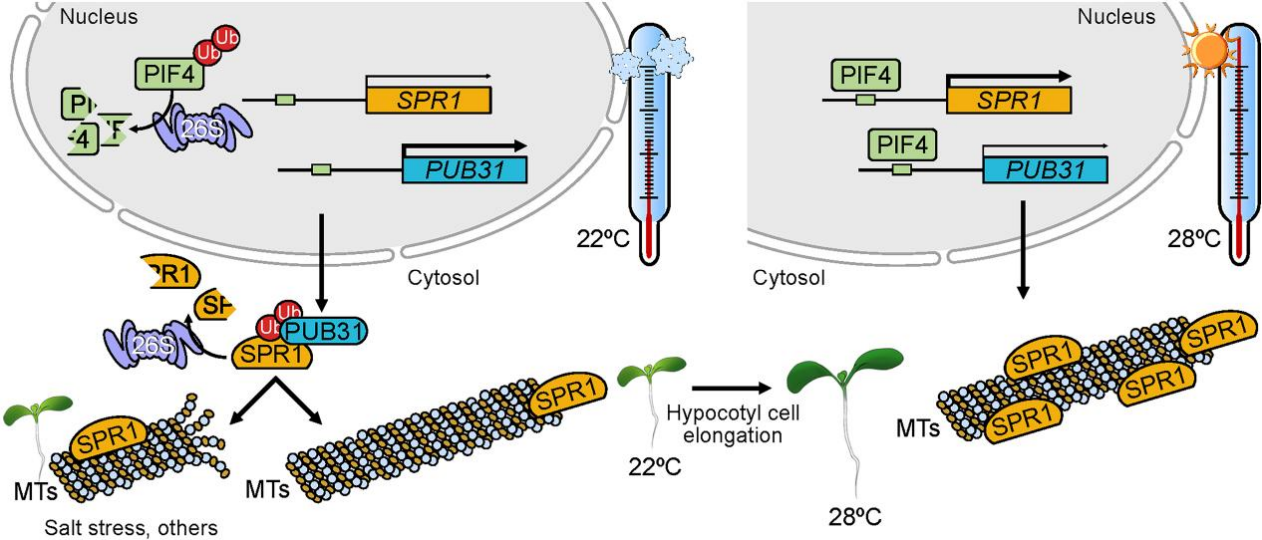
Schematic representation of SPR1 function in high temperature-induced hypocotyl elongation. At high temperatures, SPR1 is stabilized in a PIF4-dependent manner and associates with microtubules, leading to their transverse orientation and the promotion of hypocotyl elongation. The expression of *PUB31*, which targets SPR1 for 26S proteasome-mediated degradation, is blocked by PIF4 at high temperatures. Reprinted from Zhou et al. (2023), Figure 8.
